# Molecular Mechanisms and Developmental Effects of Endocrine Disruptors on Male Reproductive Health and Mitigation Strategies to Reduce Their Adverse Effects

**DOI:** 10.3390/toxics14100851

**Published:** 2026-09-24

**Authors:** Małgorzata M. Dobrzyńska, Ewa A. Jankowska-Steifer

**Affiliations:** 1 Polish Radiation Research Society, Konwaliowa 7 Str, 03-194 Warsaw, Poland; 2 Department of Histology and Embryology, Faculty of Medicine, Medical University of Warsaw, Chałubinskiego 5 Str, 02-004 Warsaw, Poland; ewa.jankowska-steifer@wum.edu.pl

**Keywords:** endocrine disruptors, male reproductive health, spermatogenesis, sperm quality, oxidative stress, epigenetic alterations, bisphenols, phthalates, pesticides, PFAS

## Abstract

Over the past decades, male reproductive health has markedly declined, as evidenced by reduced sperm concentration, impaired motility, increased morphological abnormalities, and a higher incidence of testicular disorders. Environmental factors, particularly endocrine disruptors (EDs), are increasingly recognized as important contributors due to their ability to interfere with hormonal signaling and cellular homeostasis. Mechanistically, EDs induce oxidative stress, mitochondrial dysfunction, DNA damage, endocrine imbalance, and epigenetic reprogramming. These alterations include changes in DNA methylation, histone modifications, and non-coding RNA expression that may persist beyond the period of direct exposure and potentially affect subsequent generations. In parallel, EDs disrupt the hypothalamic–pituitary–gonadal axis, impair steroidogenesis, and compromise Sertoli cell function and blood–testis barrier integrity. This part of review also discusses emerging strategies to mitigate ED-induced reproductive toxicity, including antioxidants, natural bioactive compounds, probiotics, and other interventions targeting oxidative stress, apoptosis, and endocrine dysfunction. Collectively, these findings highlight the complex molecular, epigenetic, and endocrine mechanisms underlying ED-induced male reproductive dysfunction and its potential long-term consequences.

## 1. Introduction

Since the early 1990s, a growing body of evidence has suggested a substantial decline in male reproductive health and fertility. Carlsen et al. [1] analyzed 61 papers published between 1938 and 1990 discovered that over a 50-year period (1940–1990) was reduced from 113 to 66 × 10^6^/mL and the mean sperm sample volume dropped from 3.40 to 2.75 mL. The above results were confirmed by later studies analyzing 101 papers published between 1934 and 1996 [2], whereas other studies conducted in Denmark and Sweden did not confirm the earlier finding [3,4]. However, the comprehensive meta-regression analysis of Levine et al. [5] showed a significant decline in sperm concentration between 1973 and 2011, i.e., 1.4% per year with an overall reduction of 52.4%. Simultaneously, a deterioration in sperm quality including an increased proportion of abnormal spermatozoa, decreased sperm motility and increased frequency of testicular diseases and prostate cancers were reported [1,6,7]. Moreover, significantly higher incidence of congenital malformations of genital tract in male newborns, like hypospadias or cryptorchidism were noted [1,6,8]. The above findings were also confirmed by other authors [4,9,10]. The phenomenon of decreased sperm count of contemporary men, along with its simultaneously declining quality, reduces reproductive chances. In recent decades, fertility rates have declined worldwide and in many countries have fallen below the replacement level [11]. This constitutes a significant problem from the public health perspective.

Infertility is a disease of reproductive system defined by the failure to conceive after one year or more of regular unprotected sexual intercourse [12]. Infertility affects millions of people of reproductive age worldwide. It is estimated that between 48 million couples and 186 million individuals suffer from infertility globally [13,14,15]. Infertility affects approximately 8–12% of couples with male factors contributing to 20–45% of cases [16]. In the male reproductive system, infertility is most commonly caused by problems in the ejection of semen, absence or low levels of sperm, or abnormal shape (morphology) and movement (motility) of the sperm [12].

It is believed that many environmental and man-made chemical and physical factors affect the reproductive system, which is the most sensitive target organ. These agents may reduce fertility, induce embryo loss, birth defects, and childhood cancer. Among them, the most important appear to be endocrine disruptors (EDs). Recent studies indicate that ED-induced reproductive toxicity is mediated not only by endocrine imbalance but also by oxidative stress, mitochondrial dysfunction, inflammation, DNA damage, and epigenetic reprogramming, which may affect subsequent generations. EDs may impair the function of Leydig and Sertoli cells, disrupt the blood-testis barrier, alter germ cell development, and compromise sperm production and quality [17,18].

Growing concern of the health risks associated with EDs has stimulated the development of regulatory frameworks aimed at limiting exposure. Simultaneously, increasing attention has been directed toward identifying strategies that may attenuate or counteract the adverse biological effects of these compounds.

This part of review summarizes current knowledge on the sources and classification of EDs, regulatory aspects, mechanisms underlying male reproductive toxicity, and approaches that may reduce exposure and mitigate adverse reproductive effects.

The effects of EDs on male gonads, spermatogenesis, and sperm function following prenatal and postnatal exposure, with particular emphasis on the underlying molecular and epigenetic mechanisms as well as transgenerational consequences for offspring health will be described in part two of this article.

## 2. Reference Search Strategy

This manuscript was prepared as a narrative review. The literature search was performed using the PubMed database. The names of individual endocrine-disrupting chemicals were combined with the following search terms: maternal exposure and male offspring, paternal exposure and male offspring, male germ cells exposure, spermatogenesis, sperm function, male fertility, and reproductive toxicity. Additional searches were conducted to identify publications related to endocrine disruptor regulations in the European Union as well as studies investigating strategies and compounds that may mitigate the adverse reproductive effects of EDs Experimental animal studies, in vitro studies, human epidemiological studies, relevant review articles, and regulatory documents were included. References cited in key publications were also screened to identify additional relevant studies.

## 3. Endocrine Disruptors—General Information

The term “endocrine disruptor” was used for the first time in 1991 at the Wingspread Conference Center in Wisconsin. One of the first papers on this subject was published in 1993 and reported that environmental chemicals disrupt the development of the endocrine system, and that effects of exposure during development are often permanent [19]. These compounds are also called endocrine disrupting chemicals, endocrine disrupting compounds, xenobiotics, hormonally active agents. EDs are exogenous substances structurally similar to natural estrogens and can act as estrogens, leading to adverse health effects. They mimic or interferes with the normal actions of hormones in the body. EDs can interfere with normal secretion, production, metabolism, transport or effect of a hormone leading to abnormalities in development, reproduction or homeostasis [20]. They interact with endocrine system at various levels and may affect the sexual differentiation, maturation, reproduction, growth, metabolism, digestion, cardiovascular function and excretion in human and wildlife [21]. EDs may diminish sperm quantity and quality and induce the defects in male reproductive tract. Such problems are also observed in general population [22,23]. Exposure to EDs may reduce testosterone (T) levels and impair male fertility [24,25,26]. Moreover, EDs may be responsible for endometriosis‚ early puberty‚ altered nervous system or immune function; certain cancers; respiratory problems; metabolic issues; diabetes, obesity, or cardiovascular problems; growth, neurological and learning disabilities [27].

The main route of exposure to EDs is gastrointestinal tract. People consume the contaminated food like plants, fish or water. Some EDs are components of synthetic food products for instance food coloring additives, while others migrate into food from packaging. Other route of exposure is inhalation, often during occupational exposure as waste smoke from factories or during pesticide spraying, as well as household dust and aerosol sprays. Sometimes the dermal exposure may occur. Newborns can be exposed through breast milk from the mother [28].

EDs are naturally occurring substances or synthetically produced chemicals and also and include physical agents such as artificial light or radiation that affect the endocrine system and impair human and animal health in various ways [29,30,31]. According to the World Health Organization (WHO) EDs lead to endocrine disruption in an intact organism, its progeny, or (sub) populations” [31].

EDs are present in many everyday products like, cosmetics, toilet and cleaning products, hair sprays, fragrances, shampoo, plastic and baby bottles, food containers, cookware, medical devices, toys, flame retardants, solvents and plasticizers. They may migrate from plastic containers and bottles and thereby contaminating food products and beverages.

The use of many EDs has been restricted for years. Many countries ban the use of certain drugs in selected products. Unfortunately, they are still used in other countries. Furthermore, they accumulate in the environment, thus affecting human health. Humans and animals are exposed to them constantly from fetal life through puberty to adulthood. Hereby EDs may affect not only the gametes of exposed individuals, but also their reproduction and development of the subsequent generation/generations.

The best-known EDs include among others bisphenols, phthalates, dioxins, polychlorinated biphenyls (PCBs), polybrominated diphenyl ethers (PBDEs), per- and polyfluoroalkyl substances (PFAS), nonylphenols, pesticides and parabens.

### 3.1. Bisphenols

Bisphenols (BPs) have a chemical structure consisting of two phenolic rings connected by a carbon attached to several side groups. The best-known compound is bisphenol A (BPA) and its derivatives. Several structural analogues of BPA have been identified, including bisphenol AP (BPAP), bisphenol F (BPF), bisphenol AF (BPAF), bisphenol S (BPS), bisphenol B (BPB), bisphenol Z (BPZ) and bisphenol P (BPP) [32].

BPA and its derivatives are widely used in the production of polycarbonates plastics and epoxy resins, which are used as monomers in the manufacturing of clear and tough plastic materials [33] like containers, water bottles, baby bottles, oven packs [34,35,36]. The other commonly used consumer products containing BPA are medical devices, toys, dental sealants, CDs and DVDs, computers, kitchen appliances, sports equipment. Epoxy resins are used for the manufacture of water pipes, as coatings on the inside of many food and beverage cans and containers and for manufacture of thermal paper used for sales receipts [37,38,39,40]. BPs migrate to food and beverages from food contact materials and have harmful effects on human health.

BPA enters the human body primarily through the oral route, respiratory system, and skin. It can be released from plastics used in food, beverages, and infant formula, especially if the packaging is heated, washed with detergent, or subjected to mechanical changes (crushing, stretching). Infants and young children are the most vulnerable due to their relatively low body weight and the widespread use of bottles, cups, plates, spoons, etc. made of plastics containing BPA. This compound can also be leached into patients’ saliva after receiving dental fillings [41,42]. The presence of BPA has been demonstrated in food at 4–23 µg/can, in beverages at 7–58 µg/g, and in the saliva of dental patients at 90–913 µg 1 h after filling placement [43].

BPB is used in synthesis of epoxy resins and is known as an indirect food additive used in food-contact resinous and polymeric coatings [44]. It is present in a variety of goods, including tomato peel, commercial dairy products, beverages, and canned food [45,46].

BPF due to its electrical conductivity and low viscosity is appropriate for usage in solvents. It is used in the manufacturing of epoxy resins and polycarbonate plastics, and it has supplanted BPA in industry. BPF was found for example in drinking water, drinks, dairy, and personal care products [47].

BPP is widely utilized in the manufacturing of food containers and polycarbonate plastics. It may be present in tomatoes, commercial dairy products, beverages, and cans [46].

BPS is utilized in detergents with advanced characteristics and is a component of phenolic resins and electroplating solvents, used in industry. Additionally, thermal paper and other goods bearing the designation “BPA free” are produced using BPS. Numerous commonly used products, like face cleansers, lotions, and shampoos, personal care items, cosmetics and paper goods, coins, bills, have been found to contain BPS [46].

BPAF is widely used as a coupling agent in many industries such as electronics, optical fiber and fluoroelastomers. Because of its efficiency in these uses, BPAF is a valuable substitute for BPA in the manufacturing of polycarbonate plastics and epoxy resins [48].

BPAP is used in the synthesis of polymeric materials, including polyacrylates, polyethers, and polyetherimides. It is known for its flame retardant and important plastic role. BPAP contributes to the production of plastics, rubber and other industrial products [49].

### 3.2. Phthalates

Phthalates called also phthalate esters (PAEs) are esters of phthalic acid. They are a class of dialkyl or alkyl/aryl esters of phthalatic acid (1,2-benzenedicarboxylic acid) structure in one benzene ring linked with two aliphatic ester groups, most commonly in the ortho configuration.

They are mainly used as solvents or, i.e., substances added to plastics to increase their flexibility, transparency, durability, and longevity. They are used primarily to soften polyvinyl chloride (PVC).

The majority of phthalates are used as plasticizers for the production of flexible PVC as well as in films and cable sheathing [50,51]. Moreover, phthalates are used in personal care products and cosmetics such as shampoos, soaps, eye shadow, moisturizer, nail polish and hair spray as well as in plastic items, paint, toys, wood finishers, drug coating, and particle formations [52,53,54,55,56]. Human exposure to phthalates occurs via ingestion, inhalation, and dermal absorption [57].

The following phthalates are considered the most important: di-2-ethylhexyl phthalate (DEHP), di-n-butyl-phthalate (DBP), benzyl butyl phthalate (BBP), diisononyl phthalate (DINP), di-isoheptyl phthalate (DIHP).

DEHP is a common additive used in commercial products with other plasticizers, especially is frequently added to PVC materials to enhance their flexibility as well as development resilience. DEHP is present in floors, roofing, synthetic leather products, furniture and upholstery accessories, plastic hoses, pipes and cables, tablecloths, raincoats and rain footwear, shower curtain, detergents, adhesives, varnishers, paint, toys, containers of soft drinks, food products. One of the most important sources of DEHP exposure are medical products (plastic transfusion sets, blood storage bags and tubes, intubation sets, dialysis sets, oxygen masks) [58,59].

DBP is present in cellulose acetate plastics, personal care products (e.g., nail polish, perfumes, and lacquers), varnishes and coatings (e.g., pharmaceuticals), and food packing materials [60].

BBP is used mainly as plasticizer in goods including PCV such as vinyl flooring, roofing materials, upholstery, adhesives and sealants, synthetic leather, food packaging, toys, industrial solvents, personal care products and cosmetics [60].

DINP is commonly used in a variety of products including: plasticizer in polyvinyl chloride, construction and building material, fuel and automotive products, as a flexible vinyl additive electronic materials, as cable insulation additive, as adhesives and sealants, paints and coatings, in rubber products [61,62].

DIHP is used as a specialist PVC plasticizer and in screen printing inks, in vinyl flooring, tile, carpet backing, molding and coating plastisols, and partial replacement for low molecular weight plasticizer, such as DEHP [63,64].

### 3.3. Dioxins

Dioxins i.e., polychlorinated dibenzodioxins (PCDDs) belong to a group of organic chemical compounds that are derivatives of dibenzo-1,4-dioxin (oxantrene). Their skeleton consists of one centrally located dioxane ring and two benzene rings connected to it, in which hydrogen atoms are substituted with chlorine atoms in various configurations. Dioxins have different toxicity depending on the number and position of the chlorine atoms. The crucial structures are so called lateral chlorines in positions 2, 3, 7, and 8.

There are 75 toxic congeners (i.e., chemicals entities belonging to a group of compounds with a similar chemical structure) from the PCDD group identified and described but only 7 of them have affinity for the aryl hydrocarbon receptor (AhR) and are toxic via this mechanism [65,66]. The most dangerous congener of PCDDs is 2,3,7,8-tetrachlorodibenzodioxin (2,3,7,8-TCDD or TCDD) [67].

Dioxins are persistent organic pollutants (POPs) in the environment. Dioxins are found throughout the world in the environment and they accumulate in the food chain, mainly in the fatty tissue of animals. More than 90% of human exposure is through food, mainly meat and dairy products, fish and shellfish, and via inhalation (8%) and dermal exposure (2%). Within the body, dioxins interact with the AhR [68].

Dioxins have no practical use (apart from a few documented cases of their use as poisons) and have never been intentionally produced by humans. They are mostly by-products of burning or various industrial processes [66]. The largest environmental source of these compounds is the incineration of industrial, municipal, and medical waste [69]. Dioxins are also produced as by-products in many chemical processes, such as the production of plastics and some pesticides. The main industry responsible for their emission is the pulp and paper industry, where dioxins are produced during the bleaching of pulp using chlorine. Another, equally significant source of dioxins is the metal industry, including the melting of scrap and secondary raw materials, and metal recovery [70,71].

### 3.4. Polychlorinated Biphenyls—PCBs

Polychlorinated biphenyls are a class of POPs that were historically produced as mixtures under the trade name Aroclor for use in industrial applications including hydraulic systems, dielectric fluids, flame retardants, etc. [72]. PCBs are a group of man-made organic chemicals consisting of carbon, hydrogen and chlorine atoms. The number of chlorine atoms and their location in a PCB molecule determine many of its physical and chemical properties. The first two digits usually refer to the number of carbon atoms in the phenyl rings (for PCBs this is 12). The second two numbers indicate the percentage of chlorine by mass in the mixture. For example, the name Aroclor 1254 means that the mixture contains approximately 54% chlorine by weight [73]. The PCB family comprises 209 different congeners [74].

PCBs belong to a broad family of man-made organic chemicals known as chlorinated hydrocarbons. PCBs came into widespread use in the 1930s and were banned in the 1970s. Due to their non-flammability, chemical stability, high boiling point and electrical insulating properties, they were widely used in electrical insulating and apparatus as hydraulic oils, vacuum pumps, heat-exchange systems, transformers, hydraulic and lubricating fluids, coping paper, paints, inks, plastics and rubber products, pigments, pesticides and for many other uses. PCBs were also used in building materials, as plasticizers, including the caulking used to seal joints between masonry units and around windows [75]. Because of widespread use of PCBs, their improper disposal, and semivolatile behavior, they have been released from equipment, soils, and sediments at of contaminated locations and transported up to great distances in the atmosphere before condensation and deposition [76].

PCBs can be assimilated by plants and consumed by animals and humans, resulting in their bioaccumulation in tissues and biomagnification in the trophic chain. They enter the human body through the lungs, skin and digestive tract. General population is exposed to PCBs through ingesting food, especially, animal fats, inhalation or dermal contact [77].

### 3.5. Polybrominated Diphenyl Ethers—PBDEs

Polybrominated diphenyl ethers belong to POPs. They are a group of organobromine compounds consisting of two halogenated aromatic rings. PBDEs are classified according to the average number of bromine atoms in the molecule. They are used as flame retardants, and for this reason have been used in a wide array of products, including building materials, electronics, motor vehicles, airplanes, plastics, paints, foam furniture padding, rugs, curtains, televisions, airplanes and automobiles and textiles. Theirs structure is similar to polychlorinated diphenyl ethers (PCDEs), PCBs and other polyhalogenated compounds, The life-saving benefits of fire retardants led to their popularization. PFAS bioaccumulate in the environment, water, plants and animals, from where they enter drinking water systems and the human food chain [78,79,80].

General human exposure occurs mainly through consumption of contaminated food like fish, poultry, meat and dairy products and less through inhalation and dermal absorption. Occupational exposures may occur in computer and electronic warehouses, and formulation facilities [80].

Commercial PBDEs are manufactured by bromination of diphenyl ethers resulting in a mixture of diphenyl ethers containing tetra-, penta-, hepta-, octa-, and deca-congeners in various percentages [81,82]. One of the most popular is 2,2′,3,3′,4,4′,5,5′,6,6′-decabrominated diphenyl ether (PBDE 209), which is used as flame retardant in constructed materials and it is considered less toxic than others.

### 3.6. Per- and Polyfluoroalkyl Substances—PFAS

Per- and polyfluoroalkyl substances, a group of synthetic chemicals, PFAS (R-X) are compounds consisting of a hydrophobic alkyl chain R of varying length (usually C4–C16) and a hydrophilic end group X. The hydrophobic part may be fully [R=F(CF2)n-] or partially fluorinated. The hydrophilic end group of PFAS may be n polar functional groups like carboxylate (-COO^−^), sulfonate (-SO_3_^−^), or phosphate esters, attached to a water-repellent (hydrophobic) fluorinated carbon chain, creating their unique surfactant properties [83].

PFAS are used in various industrial and consumer products to improve their physicochemical properties, such as enhanced resistance to heat, water, oil and stains [84].

There is believed that over 1400 chemicals from this group are used in over 200 different applications in industrial and household products [85]. They include coated cookware, water and stain-resistant clothes, food-handling materials, electroplating, fire-fighting foams, paints, cosmetics, detergents, home textiles, additives in chemical manufacturing, and flame retardants [85,86]. PFAS, particularly PFOS, have been used as mist suppressants that are added to metal plating and finishing baths to prevent air emissions of toxic metal fumes [73]. PFAS have been used as grease-proofing agents on food contact materials (FCM) to prevent oil, grease, and moisture from foods from leaking through the packaging. This includes coated paper and cardboard such as pizza boxes, microwavable popcorn bags, parchment paper, fast food wrappers, paper cups, pet food bags, and other items [84,87].

Human exposure to PFAS is primarily associated with ingestion and inhalation pathways, i.e., through diet, drinking water and indoor dust and air [88].

The most known PFAS are perfluorooctanoic acid (PFOA), perfluorooctane sulfonic acid (PFOS), perfluorononanoic acid (PENA) and perfluorohexane sulfonic acid (PFHxS). Their use is currently restricted or banned altogether, but they are still commonly found in the environment due to their past use.

### 3.7. Nonylphenols

Short-chain ethoxylates, carboxylic acid derivatives and nonylphenols are formed during anaerobic sewage digestion alkylphenol polyethoxylates are biodegraded by hydrolytic removal of ethoxylate groups [89]. Nonylphenols (NPs) are alkylphenols composed of phenol bearing a 9 carbon-tail. The nonyl group can be attached to the phenol ring at various locations, usually the 4- and, to lesser extent, the 2-positions, and can be either branched or linear. A branched nonylphenol, 4-nonylphenol, is the most widely produced and marketed nonylphenol [90].

Nonylphenol (NP) is widely used in such industries as chemicals, pharmaceuticals, and coatings. It is the final degradation product of alkylphenol polyethoxylates, a group of compounds extensively employed in the manufacture of lubricating oil additives, resins, plasticizers, surfactants, detergents, paints, cosmetics, and other formulated products [91,92]. NP is commonly detected in industrial wastewater-such as that from pesticide and dye production-as well as in agricultural chemicals like herbicides and in everyday products such as non-ionic surfactants, plasticizers, and antioxidants [93,94]. NP has been found in polyvinyl chloride, a material widely used in the food processing and packaging industry, and can leach into water transported through PVC pipes [90,92].

NP may enter the human body via dermal, gastrointestinal, and respiratory routes and accumulate there [95,96,97].

### 3.8. Pesticides

Pesticides are substance or mixture of substances intended for preventing, destroying, or controlling any pest, including vectors of human or animal disease, unwanted species of plants or animals, causing harm during or otherwise interfering with the production, processing, storage, transport, or marketing of food, agricultural commodities, wood and wood products or animal feedstuffs, or substances that may be administered to animals for the control of insects, arachnids, or other pests in or on their bodies [98].

The most known groups of pesticides are herbicides, insecticides, fungicides, rodenticides and pediculicides. Herbicides destroy or control weeds and other unwanted vegetation. They are commonly used on lawns. Insecticides kill or control insects. They are used in agriculture, industry, businesses, and households. Fungicides control fungi and can be used on plants or other surfaces where mold or mildew grow. They may also have a role in protecting crops. Rodenticides are pesticides which kill rodents. A pediculicide is a type of chemical compound that is neurotoxic to lice, used to treat infestations [99].

Direct exposure can occur during the application of pesticides, potentially causing immediate health effects such as respiratory problems, skin irritation, or headaches. Indirect exposure happens when pesticide residues remain on food, leach into water supplies, or accumulate in the environment, eventually affecting human health through ingestion or contact.

One of the most known and dangerous pesticide is dichlorodiphenyltriochloroethane (DDT). It belongs to organochlorines, is a historical, effective insecticide that was used after World War II to control malaria and agricultural pests, but was phased out over time due to persistence and accumulation in the environment and the food chain.

Other most dangerous is atrazine, an organic chemical compound from the triazine group. Used as a herbicide to control weeds in corn.

Widely used is glyphosate, the active ingredient in weed killer products such as RoundUp™. Glyphosate products are used weed killers worldwide in farms and in home gardens and lawns.

### 3.9. Parabens

Parahydroxybenzoic acid esters (PHBs), are known as parabens or nipagins are a group of organic chemical compounds with straight-chain alkyl group, some with branched groups and with aryl groups. Sometimes their salts are also used. Chemically, they are esters of 4-hydroxybenzoic acid (PHBA), differing in their functional group [100,101].

They are used as preservatives in cosmetics, pharmaceuticals, and food. They exhibit fungistatic activity and are effective against yeasts and molds, but have relatively low antibacterial activity.

Parabens are commonly used in cosmetics, such as face creams, body lotion and butters, shampoos and conditioners, color cosmetics, intimate hygiene products and cleansers. They are also used as preservatives in the food industry, for example, in fruit juices and marinades. In the pharmaceutical industry, as well as in cosmetics and food products, parabens act as preservatives in medications applied topically to the skin and mucous membranes [102]. Parabens are also found in hypoallergenic and children’s products and sometimes in antiperspirant and anti-dandruff agents [103].

The main reason for using parabens in cosmetics is to protect formulations from microbial growth. They are used to prevent the growth of bacteria and fungi, maintain cosmetic stability, extend the shelf life of products, especially water-based ones, prevent cosmetic spoilage during use. Parabens have also been added to foods to inhibit the growth of microorganisms. They may be present in cereals, candy, dried meats, beer, sauces, processed veggies, frozen dairy products, jams, pickles, flavored syrups, pharmaceuticals.

The most used PHBs are methyl parabens, ethyl parabens, propyl paraben, butyl parabens. In many formulations, parabens appear as a mixture, not as a single ingredient.

## 4. European Union Regulations on Endocrine Disruptors

The use of some EDs is banned in many European countries and outside of Europe. Nevertheless, many of the EDs belong to persistent environmental pollutants and are permanently present in the human environment.

### 4.1. Bisphenol A (BPA)

Directive 2009/48/EC on the safety of toys sets out the safety requirements that toys must meet in order to be placed on the market in the EU, regardless of whether they are produced in the EU or in third countries. This facilitates the free movement of toys within the single market.

However, after assessing the directive, the Commission identified a number of shortcomings in the practical application of the directive since its adoption in 2009. In particular, there is a need to ensure a higher level of protection against potential hazards associated with toys, especially those hazards posed by harmful chemicals, including BPA. The assessment also found that the directive needs to be implemented more effectively, particularly with regard to online sales. Regulation (EU) 2025/2509 of the European Parliament and of the Council on the safety of toys replaces Directive 2009/48/EC. The aim of the regulation is to ensure a higher level of toy safety across the EU, in particular by limiting hazardous chemical substances and introducing digital product passports, which facilitate supervision and control. The regulations expand the list of chemicals banned from being used in toy production. Carcinogenic, mutagenic, and reproductive-harmful substances are already on the list. Now, chemicals that disrupt the functioning of the hormonal system, respiratory system, and are harmful to the skin, as well as so-called forever chemicals, i.e., per- and polyfluoroalkyl substances, will be added. These substances are extremely difficult to break down in the natural environment and are harmful to health. The most dangerous bisphenols (desired because they give products flexibility) as well as fragrance substances in toys for the youngest children and those intended to be put in the mouth, e.g., in a teether, have also been banned [104].

In January 2011, the European Commission prohibited the use of BPA in the manufacture of polycarbonate baby bottles. BPA was banned in plastic bottles and packaging containing food for babies and children under three years from September 2018. In February 2018, the EU introduced stricter limits on BPA in food contact materials, based on the temporary tolerable daily intake established by The European Food Safety Authority (EFSA) in 2015. In 2023, the EFSA stated that current levels of exposure to BPA pose a risk to consumers in every age group, as this substance is harmful to the reproductive and hormonal systems. As a result of EFSA’s research, the tolerable daily intake (TDI) level of BPA was significantly reduced from from 50 μg/kg bw/day to a temporary TDI of 4 μg/kg bw/day. From 20 January 2025, the European Union has banned the use and trade of bisphenol A in materials that come into contact with food. The ban was introduced under Commission Regulation (EU) 2024/3190, which covers BPA, its salts, and other hazardous bisphenols and derivatives in materials intended to come into contact with food. This concerns, among others, metal coatings of food and beverage cans, reusable plastic bottles, equipment for transporting and storing food, and other kitchen accessories that come into contact with food. The ban concerns the use of BPA in the production of materials and products intended for contact with food, including adhesives, rubbers, ion-exchange resins, plastics, printing inks, silicones, varnishes and coatings [83,105].

### 4.2. Phthalates

EU law prohibits the use of phthalates as food additives. However, their use is allowed in plastics that come into contact with food, provided that they meet the conditions set by European law regarding their migration levels. In practice, their use is marginal and limited to the production of, for example, films used for packaging fat-free foods. Higher molecular weight phthalates are not used in the production of cosmetics because they do not have the appropriate properties for use in these applications. Those with lower molecular weight (i.e., di-n-butyl-phthalate DBP and diisobutyl phthalate-DIBP) were used in cosmetics; however, their use is prohibited in the EU due to legal regulations forbidding the use of substances classified as carcinogenic, mutagenic, and harmful to reproduction. The phthalates currently allowed for use is dimethyl phthalate (DMP) and diethyl phthalate (DEP).

In the European Union, the law regarding phthalates is regulated by several directives and regulations aimed at ensuring consumer safety and health protection. Regulation (EU) 2021/1297 adds long-chain perfluorocarboxylic acids C9–C14 to Annex XVII of Regulation (EC) No 1907/2006 concerning the Registration, Evaluation, Authorisation and Restriction of Chemicals (REACH) [106].

There is proposal to ban their use of in the production of consumer goods; however, due to separate legal regulations, it does not cover materials intended to come into contact with food.

As mentioned, in 2005, the EU banned the use of certain phthalates in products for infants. The new regulations will expand these restrictions, BBP, DEHP, DBP, and DIBP will not be allowed to be present in any consumer articles or in enclosed spaces in concentrations equal to or greater than 0.1% by weight in relation to a single phthalate or their mixture. The European Commission stated that “consumers may be exposed to individual phthalates or their mixtures from various sources, such as consumed food, dust, inhaled air and dust in enclosed spaces, and any articles that come into contact with the mouth, mucous membranes, and skin.

### 4.3. Dioxins and Polychlorinated Biphenyls (PCBs)

Legal regulations regarding dioxins and dioxin-like polychlorinated biphenyls are based on Directive of the European Parliament and of the Council 2014/40/EU and Commission Regulation (EU) 2022/2002. This directive harmonizes the statutory, executive, and administrative provisions of the member states regarding the production, presentation, and sale of tobacco products and related products. Commission Regulation (EU) 2022/2002 amends Regulation (EC) No 1881/2006 concerning the maximum permissible levels of dioxins and dioxin-like PCBs in certain foodstuffs. These regulations aim to ensure food safety and prevent food contamination by dioxins and PCBs. In cases of problems with the presence of these substances in food, legal regulations allow for their assessment and control, as well as the introduction of protective measures, such as limiting the consumption of food affected by contamination 2014/40/EU [107,108].

Council Directive 96/59/EC of 16 September 1996 regulates the disposal of PCB and polychlorinated terphenyls (PCT). In the context of legal regulations, Council Directive 76/403/EEC concerns the disposal of PCBs and also requires a gradual move towards a total ban on their use. PCBs are toxic substances that can affect human health and the environment, which is why proper disposal and decontamination measures are important [109,110].

At the end of 2004, products containing penta- and octa-congeners of PBDE were withdrawn from the market because studies showed their presence in human tissues and breast milk. Deca-derivatives are still used because a number of toxicological studies have not found their negative effects on human health, while as flame retardants they increase safety in the event of a fire [111].

### 4.4. Per- and Polyfluoroalkyl Substances (PFAS)

The European Union is introducing increasingly strict regulations limiting the use, emission, and production of per- and polyfluoroalkyl substances. There are several key legal acts in force in the EU regarding PFAS. Regulation (EC) No 1907/2006 contains criteria for “very persistent” substances and requirements for the registration, evaluation, and restriction of chemical substances, including PFAS. Regulation (EU) 2019/102 prohibits the use of the most harmful PFAS, including PFOS and PFOA, and introduces other restrictions. Regulation (EU) 2025/1988 amends Annex XVII to Regulation (EC) No 1907/2006, which concerns PFAS. Regulation (EU) 2021/1297 adds long-chain perfluorocarboxylic acids C9–C14 to Annex XVII of REACH. The proposal submitted by the European Chemicals Agency (ECHA) introduced broad restrictions on the use of PFAS and was aimed at protecting human health and the environment from PFAS-related risks, while contributing to the gradual elimination of these substances from consumer products in the EU [112,113,114,115].

### 4.5. Nonylphenol

In the European Union, nonylphenol is regulated under REACH (Regulation EC) No. 1907/2006. Following a public consultation, nonylphenol was excluded from the scope of the restriction proposal because it is not intentionally used in textile processing. Nonylphenol ethoxylates, however, are subject to restrictions, including a ban on their marketing above specified concentration limits. Under current regulations, nonylphenol and nonylphenol ethoxylates cannot be placed on the EU market under specified conditions. These regulations mainly apply to textile products that can be washed in water at concentrations of at least 100 mg/kg (0.01% by weight). These values are defined in the Annex XV dossier [112].

Additionally, recent EU cosmetics regulations introduce limits and conditions for the use of nonylphenol, which is classified as a CMR substance (carcinogenic, mutagenic or toxic to reproduction). The new regulations aim to enhance consumer protection while allowing manufacturers to use certain substances within specified concentrations and products.

### 4.6. Pesticides

DDT was one of the first pesticides to be banned after years of widespread use and recognition as an effective insecticide. It was used during World War II to combat lice and fleas and protect against typhus. Later, thanks to DDT, malaria was eradicated from Europe and North America, and its occurrence was significantly reduced in Africa and South America. However, over time evidence began to emerge that DDT has serious negative effects on the environment and human health. DDT is a persistent chemical compound that accumulates in the food chain, leading to bioaccumulation and biomagnification. High concentrations of DDT were found in the bodies of predators at the top of the food chain, which had catastrophic effects on many species of birds of prey. Furthermore, there is evidence suggesting that DDT may be carcinogenic and may cause hormonal disruptions in humans. As a result of these discoveries, many countries decided to ban the production and use of DDT in the 1970s. These restrictions were further reinforced by international agreements, including the Stockholm Convention on Persistent Organic Pollutants, adopted in 2001 and ratified by numerous countries, which committed to eliminating or restricting the production and use of persistent hazardous chemicals. The ban was aimed at protecting public health and preserving the ecological balance of our planet [116,117].

Other banned pesticides, including neonicotinoids, carbendazim, benomyl, glufosinate, methyl thiophanate, acephate, atrazine, clethodim, mancozeb, omethoate have a documented negative impact on human health and ecosystems. They can cause water and soil contamination, as well as contribute to the decline in populations of pollinating insects, such as bees, by weakening their nervous systems, causing disorientation, and reducing the reproductive capacity of queen bees. Residues of these substances in food can increase the risk of diseases in humans, and the global health and environmental costs associated with their use are estimated at USD 12.7 trillion annually, of which 73% are health costs. Although the EU bans the use of many pesticides on its own market, it allows the import of food produced using them, e.g., soy from Brazil and the USA or rapeseed from Australia and Canada. These products are primarily used to the feed industry and subsequently reach consumers in the form of meat, milk, or eggs, without mandatory labeling of genetically modified organism (GMO) origin or pesticide-related production practices. In the context of food safety, pesticide levels are regulated by various laws that define the maximum allowable levels of pesticide residues in food. Regulation (EC) No 396/2005 establishes the maximum allowable levels of pesticide residues in food and feed of plant and animal origin and on their surface. Regulation (EU) 2025/158 amended Annexes II, III, and IV to Regulation (EC) No 396/2005 by introducing revised maximum residue levels for acetamiprid in fruits and vegetables. Commission Implementing Regulation (EU) 2024/989 establishes additional revisions to pesticide residue limits, applicable from 8 April 2024. These regulations aim to ensure food safety and uniform application of the rules in the European Union. The new standards are the result of risk assessments by the EFSA and new toxicological data (Regulation [118,119].

### 4.7. Parabens

In the European Union, following a safety review conducted by the Scientific Committee on Consumer Safety (SCCS), of certain parabens have been classified as EDs. Consequently, the use od parabens in cosmetics has been restricted or banned, depending on their classification.

## 5. Mechanisms of Male Reproduction and Their Susceptibility to Disruption

### 5.1. Oxidative Stress as a Major Mechanism of Sperm Damage

EDs may kill directly germ cell or affect them by several ways. In addition to direct cytotoxicity, EDs may also disturb cellular redox balance, mitochondrial function, and intracellular signaling pathways, thereby amplifying oxidative damage in germ cells [17]. As was mentioned before, diminishing of sperm quality is caused by oxidative stress since male gametes are vulnerable to oxidative damage by free radicals [120,121,122]. Cell membrane of spermatozoa is rich in polyunsaturated fatty acids, in particular docosahexaenoic acid. Polyunsaturated fatty acids include of unconjugated double bonds containing electrons, which may be donated to reactive oxygen species (ROS), causing the formation of lipid peroxides. As the result, the fluidity of the sperm cell membrane is impaired, leading to decrease in sperm viability and motility [123,124,125]. Furthermore, mitochondria located in the sperm midpiece are highly sensitive to ROS overproduction, and their dysfunction leads to loss of membrane potential and reduced ATP generation, which further compromises sperm motility and viability [126]. Sperm viability is impaired by the transformation of essential membrane proteins and an uncommon acrosome reaction that cause a diminished ability of spermatozoa to connect with oocyte. Sperm motility is touched in account of a attenuation in axonemal protein phosphorylation. Moreover, ROS play a dual role in sperm physiology, as they are required at physiological levels for processes such as capacitation and protein phosphorylation, but their excess disrupts intracellular signaling pathways and calcium homeostasis [127,128]. ROS may also prompt DNA injury in male gametes. This take place through a direct attack on the bases or the phosphodiester backbones, leading to destabilization of the DNA particle and the generation of anomalies. DNA damage may cause faulty fertilization, decreased implantation and poor embryo development, and afterwards elevated mortality and risk of developing cancer. Due to the highly condensed chromatin structure and the absence of efficient DNA repair mechanisms in mature spermatozoa, such damage is largely irreversible and can be transmitted to the oocyte, where incomplete or faulty repair may result in de novo mutations and adversely affect early embryonic development [129]. Otherwise, strongly injured spermatozoa may undergo apoptosis, leading in consequence to low sperm count [123,124,125,130,131,132]. Importantly, even when fertilization occurs, oxidative damage to sperm DNA and associated epigenetic alterations may impair early embryonic development and contribute to increased disease susceptibility in the offspring [133].

Importantly, it is not solely the absolute increase in ROS levels but rather the disruption of redox homeostasis that determines the extent of cellular and epigenetic damage. Environmental toxicants, including EDs such as bisphenol A, phthalates, and heavy metals, contribute to this imbalance by enhancing oxidative stress and weakening antioxidant defenses [134]. As both a major source and target of ROS, mitochondria play a central role in this process, and their dysfunction may further promote oxidative stress while impairing pathways involved in germ cell survival, differentiation, and epigenetic programming.

#### 5.1.1. ROS, Epigenetic Alteration and Testicular Dysfunction

Elevated ROS in testis may be associated with dysfunction in site-specific hypermethylation through the upregulation of DNA methyltransferases (DNMTs) or the formation of complexes involving these enzymes [135,136,137]. In addition, ROS-induced oxidative DNA lesions, such as the formation of 8-oxo-2′-deoxyguanosine (8-oxo-dG), may promote mutagenic base mispairing and interfere with base excision repair mechanisms, further contributing to genomic instability. Importantly, beyond its mutagenic potential, 8-oxo-dG has also been implicated in the epigenetic regulation of gene expression, suggesting that oxidative DNA damage may simultaneously affect genome integrity and transcriptional programming [138,139]. An increase in seminal ROS production and reduction in antioxidant enzyme are believed to be associated with of sperm alterations, like chromosomal abnormalities, induction of micronuclei, changes in sperm membrane potential, increased level of apoptosis and single or double DNA damage, which may lead to increased risks of implantation failure or miscarriage. The main targets of ROS-induced damage in the testis include Leydig cells, seminiferous tubules, and spermatozoa. By disrupting Leydig cell function, ROS reduce testicular steroidogenesis and T synthesis, thereby impairing spermatogenesis and potentially contributing to male infertility [140]. High ROS level in the semen provoke sperm irregularities, leading to teratozoospermia and decreased fertility [141,142]. Importantly, mitochondria represent both a major source and a primary target of ROS in spermatozoa; oxidative damage to mitochondrial membranes results in loss of membrane potential (ΔΨm) and impaired sperm motility [143,144]. Endocrine disruptors may increase oxidative stress by downregulating the generation of antioxidant enzymes, resulting in male infertility by disrupting the cell junction and adhesion between Sertoli–Sertoli cells and/or Sertoli–germ cells via the phosphatidylinositol 3-kinase/c-Src/focal adhesion kinase signaling pathway [145]. In addition to DNA methylation, oxidative stress can also affect post-translational histone modifications (e.g., H3K4me3 and H3K27me3) by modulating the activity of enzymes responsible for histone demethylation and deacetylation, thereby altering chromatin accessibility and transcriptional programming [146].

Furthermore, ROS have been shown to modify the profile of small non-coding cRNAs (ncRNAs) of sperm, including miRNAs and tsRNA-derived fragments, which are involved in early embryonic gene regulation and may also contribute to the epigenetic inheritance of environmental influences [147].

Alterations in DNA integrity and epigenetic programming caused by ROS may persist in mature sperm and be transmitted to the embryo, thus contributing to altered developmental course and potentially to transgenerational effects [133]. Therefore, oxidative stress should not be viewed solely as a cause of cellular injury but also as a potential mediator linking environmental exposure with epigenetic inheritance through sperm-borne molecular information.

The major ROS-mediated mechanisms linking oxidative stress with epigenetic alterations, sperm dysfunction, and potential transgenerational inheritance are summarized in Figure 1.

#### 5.1.2. Structural and Epigenetic Organization of the Sperm Genome

The sperm genome is distinct from that of somatic cells and the oocyte due to its unique structural organization and biological function. During spermatogenesis, the bulk of histones are replaced by protamines to maximize chromatin compaction and ensure structural stability of the paternal genome; however, a residual ~1–15% histone fraction is selectively retained across mammals. It is important to emphasize that this highly compressed chromatin architecture, combined with the limited capacity of mature sperm to repair DNA, makes the paternal genome particularly susceptible to oxidative stress-induced damage. At the same time, the extensive chromatin remodeling that occurs during spermatogenesis creates multiple windows of susceptibility during which environmental contaminants may interfere with the establishment and maintenance of epigenetic marks [148].

Importantly, these histone-retaining nucleosomal regions in sperm chromatin are not randomly distributed: they are enriched at promoters with unmethylated CpG islands, regulatory elements such as enhancers and super-enhancers, as well as at specific genomic features including telomeres, transcription start sites (TSSs) and even gene-poor (desert) regions [149]. The selective localization of retained histones to regulatory regions may create epigenetically active domains that are particularly sensitive to environmental stressors, including oxidative damage and endocrine-disrupting chemicals [150]. These retained nucleosomes carry distinctive post-translational modifications (PTMs)—notably H3K4me3 and H3K27me3—that are poised to influence zygotic genome activation (ZGA) and early lineage decisions, positioning retained histones as strong candidates for paternal epigenetic inheritance [148]. Consequently, environmentally induced alterations in these histone marks may affect embryonic gene regulation long after fertilization, despite the extensive epigenetic reprogramming that normally occurs during early development.

In parallel, alterations in protamine content or in the protamine 1/protamine 2 ratio have been associated with decreased chromatin stability, increased DNA fragmentation, and enhanced susceptibility to oxidative stress, highlighting the critical role of proper chromatin packaging in maintaining sperm genome integrity [151].

Moreover, spermatozoa deliver a specialized repertoire of small RNAs, including miRNAs, piRNAs, and tsRNAs, which—together with DNA methylation patterns and the retained histone fraction—form a distinctive paternal epigenetic signature transmitted to the oocyte at fertilization [152,153].

#### 5.1.3. Paternal Epigenetic Information and Susceptibility to Endocrine Disruptors

This triad of DNA methylation, histone-based chromatin signals, and small RNAs is now considered the primary carrier of paternal epigenetic information that shapes early embryonic programming. Recent reviews and multi-omics animal studies demonstrate that these epigenetic layers are particularly susceptible to modulation by EDs, highlighting their importance not only in normal reproduction but also in environmentally induced developmental perturbations. These epigenetic layers do not function independently but form an interconnected regulatory network in which oxidative stress, mitochondrial dysfunction, and endocrine signaling may converge to modify sperm-borne information transmitted to the next generation [150].

EDs may interfere with epigenetic programming through multiple mechanisms, including altered DNA methyltransferase activity, disruption of histone-modifying enzymes, and changes in sperm small RNA profiles, thereby perturbing the coordinated regulation of gene expression in early embryos [154].

Notably, a growing body of evidence suggests that environmentally induced epigenetic alterations may persist across generations, supporting a role for ED mediated epigenetic inheritance in the developmental origins of health and disease. Although extensive epigenetic reprogramming occurs after fertilization, certain genomic regions and epigenetic marks can escape this process, enabling the persistence and transmission of environmentally induced paternal epigenetic modifications to the embryo. This concept provides a mechanistic framework linking paternal exposure to EDs with altered offspring phenotype and supports the growing evidence for environmentally mediated intergenerational and transgenerational inheritance [155].

### 5.2. Hormonal Regulation and Disruption in the Male Reproductive System

#### 5.2.1. Physiological Role of Estrogens in Male Reproduction

Although traditionally considered female hormones, estrogens are essential regulators of male reproductive physiology. Estrogens are produced from androgens through the activity of the aromatase encoded by CYP19 gene. The presence of estrogen receptors (ERα and ERβ) and aromatase has been found in the reproductive, cardiovascular system, adipose tissue, muscle cells, brain, bones, and lung of males. Aromatase, plays an crucial role in the conversion of androstenedione and testosterone into estrone and estradiol, and is expressed in Leydig cells, germ cells, and epididymal sperm. The absence of aromatase induces post-meiotic defects, characterized by increased apoptosis and reduced fertility, Estrogens exert direct regulatory effects on spermatogenesis. and the development of male reproductive organs. Studies in ERα knockout models have shown that the efferent ductal epithelium, which connects the testis to the initial segment of the epididymis, fails to absorb fluid properly This leads to backpressure atrophy in the testes and disrupts the spermatogenesis. They are responsible for the development of the reproductive system during the prenatal period of life. Estradiol acts synergistically with follicle-stimulating hormone (FSH) to promote maturation of seminiferous tubules and initiation of spermatogenesis. Subsequently, estrogens participate in maintaining spermatogenesis through the regulation of Leydig cell steroidogenesis, Sertoli cell function, and germ cell differentiation. Estrogens are also responsible for fluid resorption in efferent ductules and the epididymis, thus influencing sperm concentration. Furthermore, they regulate key processes such as sperm capacitation and the acrosome reaction, as well as sperm survival. In addition, they stimulate bone growth and mineralization, inhibit long bone growth in the final period of puberty, prevent the development of abdominal obesity and lipid disorders, and have a cardioprotective effect [156,157,158,159,160,161,162].

#### 5.2.2. Mechanisms of Estrogen Signaling

Female sex hormones can directly or indirectly affect gene expression through nuclear ERα and ERβ and influence cells through estrogen receptors present in the cell membrane (non-genomic effects). Natural estrogens and endocrine like compounds can only affect cells with estrogen receptors. The most thoroughly understood action of estrogens involves regulating the expression of specific genes. Binding of the hormone to the estrogen receptor causes its phosphorylation, a change in conformation, and binding to a specific region of DNA. The estrogen–receptor complex acts as a transcription factor that enhances or inhibits gene expression. It can also stimulate the production of other transcription factors that influence the transcription of certain genes. These genomic and non-genomic signaling pathways allow precise regulation of gene expression in a cell-type-specific manner [163,164].

#### 5.2.3. Mechanisms of Endocrine Disruptor Action on Hormonal Pathways

One of the most common mechanisms of action of EDs is hormonal mimicry, the structural similarity of endocrine-like compounds, especially to steroid sex hormones, allows them to easily bind to estrogen as well as to androgen receptors, primarily through competitive mechanisms. A ligand with a chemical structure similar to estradiol binds to the estrogen receptor and acts as an agonist or antagonist, depending on the biological context. EDs can block estrogen receptors or, through the receptor, inhibit the action of natural hormones. They can also affect the synthesis, metabolism, or elimination of hormones from the body. Endocrine disruptors may influence hormone bioactivity, for example by altering the concentration of sex hormone-binging protein, and can also directly affect gene expression. Moreover, they can disrupt hormonal balance and affect the activity of hypothalamus or pituitary gland, thereby interfering with the hypothalamic–pituitary–gonadal (HPG) axis. This dual ability to mimic or inhibit endogenous hormones results in dysregulated signaling and impaired reproductive homeostasis [165]. The major molecular and cellular mechanisms involved in endocrine disruptor-induced impairment of male reproductive function are schematically illustrated in Figure 2.

#### 5.2.4. Gonadal Targets and Reproductive Consequences of Endocrine Disruptors

In the gonads, endocrine-like compounds, dysregulate key steroidogenic enzymes, including 3β-hydroxysteroid dehydrogenase/∆-5-4 isomerase (3β-HSD), cytochrome P450 enzymes such as CYP11A1 and CYP17A1, as well as 17β-hydroxysteroid dehydrogenases (17β-HSD) and the secretion of steroid hormones, the localization and expression of ERα in Leydig cells, the expression of the androgen receptor and proteins in the gonad, and modulate the acrosomal reaction [166,167,168,169,170].

Additionally, EDs can alter aromatase activity, thereby disturbing the balance between androgens and estrogens in the testicular microenvironment [171].

Endocrine disruptors also impair the integrity of the blood–testis barrier by disrupting Sertoli–Sertoli and Sertoli–germ cell junctions, leading to altered germ cell development [172]. In this way, EDs may reduce male fertility, cause erectile dysfunction, induce testicular and prostate cancers, as well as abnormal sexual development, alteration in the pituitary and thyroid gland functions, immune suppression, and neurobehavioral effects [173]. Altogether, these findings indicate that EDs target multiple cellular and molecular pathways within the gonads, resulting in cumulative and potentially irreversible impairment of male reproductive function.

Collectively, these alterations may be interpreted within the framework of testicular dysgenesis syndrome (TDS), a developmental condition linked to impaired fetal testicular development and manifesting as multiple reproductive abnormalities.

## 6. Actions Recommended to Avoid or Reduce the Adverse Effects of Endocrine Disruptors

Many substances, both natural and man-made, can cause endocrine disorders. Because EDs come from many different sources, exposure to these substances is associated with the air we breathe, the food we consume, and the water we drink. EDs can also enter the body through the skin.

Many EDs are used as pesticides, plasticizers, flame retardants, industrial chemicals, and components of consumer products. EDs are often used in industry, which is why they can be found in food, including food products, packaging, clothing, furniture, and electronic equipment. Especially, they are present in widely used everyday objects and products like cookware, plastic bottles and containers used for food and beverages, scented candles and fragrances, cosmetics, cleaning supplies, hair sprays and shampoos. It is important to increase awareness of the surroundings and avoid contact with EDs, which may be contained in consumer products such as plastics, paints, detergents, cosmetics. Dietary intake remains a primary pathway, particularly through the consumption of processed or packaged foods and beverages that have come into contact with plasticizers or food-contact chemicals [174,175]. To avoid exposure to endocrine-disrupting compounds, it is worth limiting the consumption of food containing such compounds. Environmental exposure is also significant, especially through contaminated air, dust, or water, and is influenced by the indoor and outdoor environments [176,177]. Environmental policies aimed at reducing the use of plasticizers, pesticides, and POPs may contribute to lowering population exposure to EDs [178].

During everyday life, humans are exposed to complex mixtures of EDs rather than isolated compounds. They are simultaneously exposed to multiple chemicals coming from food, consumer products, indoor environments, occupational settings, and environmental contamination. Such combined exposures may produce additive, synergistic, or antagonistic biological effects, even when individual compounds are present at relatively low concentrations. Consequently, evaluating single chemicals in isolation may underestimate the complexity of real-world endocrine disruption and complicate the interpretation of interventions [179].

Given the widespread exposure to EDs and their documented adverse effects on reproductive health, increasing attention has been directed toward identifying compounds that can prevent, attenuate, or counteract ED-induced toxicity. Such protective agents, including antioxidants, natural bioactive molecules, and micronutrients, may mitigate the detrimental effects of EDs by modulating oxidative stress, apoptosis, inflammation, and steroidogenic pathways.

Most protective interventions investigated to date exert their beneficial effects through a limited number of common mechanisms, including reduction of oxidative stress, suppression of apoptosis, preservation of steroidogenesis, maintenance of blood–testis barrier integrity, and restoration of spermatogenesis. The main classes of protective compounds and the molecular mechanisms underlying their beneficial effects against endocrine disruptor-induced male reproductive toxicity are presented in Figure 3.

### 6.1. Antioxidants and Micronutrients

Antioxidants and micronutrients constitute the most extensively investigated group of compounds mitigating endocrine disruptor-induced male reproductive toxicity. Their beneficial effects are primarily associated with attenuation of oxidative stress, enhancement of antioxidant defense systems, reduction of apoptosis, preservation of steroidogenesis, and improvement of spermatogenesis and sperm quality.

Chinese study by Gao et al. [180] reported that although co-exposure to a range of EDs is mainly associated with deteriorated sperm quality and less with sperm quantity, high seminal omega-3 polyunsaturated fatty acid status, i.e., elevated docosapentaenoic acid (DPA) level, might be protective against such effects.

In rats exposed to BPA, vitamin E preserved seminiferous tubule structure and reduced the presence of immature germ cells. At the highest dose, *Myrtus communis* essential oil (EOMC), combined with BPA, improved sperm parameters, restored testicular histology, preserved membrane phospholipid composition, and normalized levels of steroid hormones including testosterone, estradiol, progesterone, and cortisol [181]. Co-administration with vitamin C reversed the effect of bisphenol A-induced oxidative stress in epididymal sperm and epididymis [182]. Deterioration of sperm motility and morphology induced by a relatively large dose of BPA during neonatal exposure was inhibited by supplementation with vitamin A [183].

Wu et al. [184] showed that a significant increase in TBARS levels and a decrease in SOD activity, as well as an increase in DNA migration within male germ cells and γH2AX foci formation on the autosomes of pachytene spermatocytes induced by BPA administration in Sprague-Dawley rats, were significantly alleviated by melatonin pretreatment. Similarly, co-administration of BPA and melatonin decreased oxidative stress by modulating the levels of glutathione, superoxide dismutase, and catalase as well as malondialdehyde and hydrogen peroxide (H_2_O_2_) concentrations in the testes and sperm. Moreover, melatonin sustained Bcl-2 expression and testosterone levels, controlled apoptosis, ameliorated histopathological changes, increased the percentages of seminal haploid germ cells, and protected the sperm chromatin condensation process, indicating appropriate spermatogenesis with production of functional sperm [185]. Melatonin reduced the damage induced by BPA to both epididymal tissue and sperm quality, although it did not prevent it completely [186].

Melatonin (10 mg/kg BW/alternate day) pretreatment in BPS-exposed adult male golden hamsters (*Mesocricetus auratus*) resumed normal testicular histoarchitecture and sperm quality and decreased testicular oxidative load, as evidenced by enhanced antioxidant enzyme (SOD and catalase) activities and decreased lipid peroxidation (LPO) levels. Melatonin stimulated the testicular antioxidant proteins Nrf-2/HO-1 and SIRT-1/FOXO-1 and reduced inflammatory proteins NF-kB/COX-2 expression to counteract BPS-induced testicular damage. Moreover, melatonin increased testicular cell proliferation (PCNA), survival (Bcl-2), and gap-junction protein expression (connexin-43), and decreased apoptosis (caspase-3) [187]. Melatonin supplementation to BPS-exposed lactating mothers improved testicular histoarchitecture in offspring, enhanced testicular antioxidant status, and modulated the expression of redox/survival and blood-testis barrier markers, promoting germ cell proliferation [188].

Melatonin reversed changes in the gonadosomatic index along with sperm quality and quantity induced by DEHP. Moreover, melatonin mitigated testicular oxidative stress, restored serum testosterone levels, and reversed DEHP-induced protein expression of C/EBP homologous protein (CHOP), 78 kDa glucose-regulated protein (GRP-78), and caspase-12 in testicular tissue [189].

Zinc and selenium exerted protective effects against BPA-induced male reproductive toxicity. Co-administration of Zn and Se ameliorated BPA-induced gonadal damage in rats, as evidenced by reduced serum SGPT, SGOT, and blood urea nitrogen levels, decreased testicular and epididymal tissue injury, DNA damage, apoptosis, and expression of 8-OHdG, γ-H2AX, and NF-κB, together with increased serum testosterone and catalase levels. Similarly, selenium supplementation attenuated BPA-induced oxidative damage by reducing oxidized phospholipid levels and enhancing antioxidant enzyme activities, resulting in improved testicular function and epididymal sperm parameters [188,189]. Collectively, these findings highlight the antioxidant and gonadoprotective properties of Zn and Se in mitigating endocrine disruptor-induced testicular dysfunction [190,191].

Simultaneous supplementation with alpha-tocopherol and ascorbic acid restored Aroclor 1254-induced decreases in epididymal sperm motility and count to normal ranges. Alpha-tocopherol and ascorbic acid exerted protective effects on sperm by inhibiting PCB-induced ROS generation [192].

Vitamin E supplementation attenuated TCDD-induced reproductive toxicity by restoring GnRH, LH, and FSH levels, reversing the decline in T concentrations and sperm count, and ameliorating testicular structural alterations [193].

The study of Khan and Sinha [194] demonstrated a dose-dependent amelioration by vitamin C in the primary spermatocytes of Swiss albino mice exposed to phosphamidon. Similarly, vitamin C ameliorated sperm count and morphology diminished by exposure to phosphamidon, endosulfan, and mancozeb [195]. Endosulfan alone significantly reduced sperm count and motility and increased abnormal sperm morphology. Ascorbic acid treatment produced significant improvement, reaching control values in most parameters except sperm motility [196]. Likewise, L-ascorbic acid dose-dependently attenuated endosulfan-induced reductions in sperm count and motility and decreases in sperm morphology quality, although values did not fully return to control levels [197].

Co-treatment of malathion-exposed rats with vitamins E and C exerted protective effects on sperm count, sperm motility, and abnormal sperm numbers, although not on plasma FSH, LH, and T levels [198]. Similarly, addition of vitamins C and E alone or especially in combination significantly improved sperm motion, sperm function, and DNA integrity in human sperm exposed to cypermethrin in vitro [199]. In caprine testicular cells, vitamins C and E ameliorated cypermethrin-induced germ cell apoptosis and thereby prevented spermatogonial cell degeneration and male infertility [200].

N-acetylcysteine (NAC) supplementation ameliorated the toxic testicular effects of glyphosate, decreased apoptosis rates and oxidative stress in caprine germ cells [201]. The triple combination of N-acetylcysteine, parthenolide, and 3-methyladenine markedly restored cell proliferation, decreased BBP-induced apoptosis and autophagy, and restored mTOR phosphorylation in male germ cells [202]. Other studies by Bhardwaj et al. [203,204] showed that NAC mitigated glyphosate-induced cytotoxicity in testicular cells and reduced apoptosis and oxidative stress. Similarly, NAC reduced malathion-induced reproductive toxicity by enhancing cell viability, diminishing apoptosis and DNA fragmentation, reducing oxidative stress parameters, and increasing ferric reducing antioxidant power in testicular germ cells.

Glutathione co-administration mitigated mancozeb-induced reproductive toxicity by increasing serum levels of FSH, LH, and T, while restoring testicular lactate dehydrogenase (LDH), acid phosphatase (ACP), and alkaline phosphatase (ALP) activities. These effects were accompanied by improved 3β-HSD activity, increased sperm viability, and a reduced incidence of abnormal spermatozoa [205].

Co-administration of α-tocopherol improved SOD, GPx, and glutathione S-transferase (GST) activities, increased the number of spermatozoa and spermatids, and improved most sperm parameters and testicular histoarchitecture compared with the effects of flubendiamide alone [206].

Benfotiamine, a fat-soluble derivative of vitamin B1, ameliorated the deleterious effects of cyfluthrin on sperm count and sperm quality in prepubertal Wistar rats [207].

Coenzyme Q10 co-treatment ameliorated the harmful effects induced by glyphosate by restoring redox balance, normalizing signaling pathways, improving sperm quality, and correcting hormonal alterations [208].

Taken together, the available evidence indicates that antioxidants and micronutrients consistently improve sperm quality, testicular histology, steroidogenesis, and reproductive hormonal balance in experimental models exposed to EDs. Their protective effects are predominantly mediated through reduction of oxidative stress, enhancement of endogenous antioxidant defense systems, and suppression of apoptosis.

### 6.2. Polyphenols, Flavonoids and Related Phytochemicals

Polyphenols, flavonoids and related phytochemicals represent another large group of compounds capable of attenuating endocrine disruptor-induced reproductive toxicity. Their protective effects are generally attributed to antioxidant, anti-inflammatory, anti-apoptotic and pro-steroidogenic activities, resulting in improved spermatogenesis, sperm quality and testicular function.

Flavone morin (MRN) dose-dependently improved BPA-induced impairment of sperm quality by preventing oxidative stress in rats [209].

The recent study of Waleed et al. [210] showed that resveratrol improved sperm quality, testicular histopathology and reduced DNA fragmentation induced by BPA in Wistar rats.

The toxicological impact of BPA on sperm viability and motility was ameliorated by co-incubation with *Eruca sativa* aqueous extract (ESAE) at a low concentration (15.62 μg/mL) in a human spermatozoa model [211]. These results were confirmed in a subsequent study, where ESAE co-administration at low doses improved sperm count and quality as well as histological and biochemical parameters altered by BPA in male Wistar rats [212].

Ellagic acid (EA, C_14_H_6_O_8_), a naturally occurring phenolic compound, reversed the DEHP-induced reduction in numbers of spermatogonia, primary spermatocytes and Sertoli cells, as well as reductions in sperm vitality and progressive motility in mice. Moreover, EA inhibited DEHP-induced alterations in serum hormone levels [213].

Chrysin (CHR), a flavonoid present in honey and propolis, increased sperm numbers reduced by DEHP exposure and ameliorated DEHP-induced hormonal disturbances, In particular testosterone depletion, as well as histological alterations of the testis [214].

Naringin (NG), a dietary bioflavonoid administered one hour before DBP exposure, mitigated dose-dependent reproductive dysfunctions induced by DBP, as evidenced by significant increases in serum testosterone level, sperm motility, count and viability, together with marked improvement in oxidant/antioxidant status and testicular histology [215].

Farombi et al. [216] demonstrated that kolaviron, a biflavonoid isolated from *Garcinia kola* seeds, and curcumin from *Curcuma longa* ameliorated DBP-induced alterations in biochemical and spermatological parameters as well as testicular histology. Their findings indicated that both compounds protect against oxidative testicular damage induced by DBP.

Combined treatment of TCDD with lycopene (LC) or EA prevented the development of TCDD-induced damage in sperm quality, testicular histology and lipid peroxidation. Both compounds protected against functional and structural damage as well as apoptosis in spermatogenic cells [217].

Ijaz et al. [218] demonstrated that nobiletin (NOB), a polymethoxyflavone, reversed reproductive toxicity induced by nonylphenol. NOB restored activities of CAT, SOD, GPx and GSR while reducing TBARS levels. Moreover, NOB increased FSH, LH, plasma testosterone, daily sperm production, epididymal sperm count, viability, motility, expression of steroidogenic enzymes (StAR, 3β-HSD and 17β-HSD), anti-apoptotic Bcl-2 expression, and the number of spermatogenic cells. In contrast, sperm abnormalities, apoptotic markers (Bax and caspase-3), and histopathological damage were significantly reduced.

Amentoflavone (AMF) displayed considerable potential to attenuate NP-induced testicular damage in rats, including dead spermatozoa, abnormalities of the sperm head, tail and midpiece, and disturbances in antioxidant enzyme activities. The protective effects of AMF were associated with its anti-apoptotic, antioxidant, anti-inflammatory and pro-steroidogenic properties involving modulation of the Nuclear factor erythroid 2-related factor 2 (Nrf2)/Kelch-like ECH-associated protein 1 (Keap1) axis and restoration of steroidogenesis [219].

Gules et al. [220] demonstrated that LC and gallic acid (GA) supplementation significantly improved seminiferous epithelium height, activities of testicular SOD, CAT and GSH, and sperm viability in comparison with 4-nonylphenol exposure alone. Additionally, LC and GA reduced histopathological lesions, including germinal epithelial degeneration, vacuolization, immature germ cells in the tubular lumen, interstitial congestion and the percentage of abnormal spermatozoa.

Curcumin administration ameliorated lindane-induced reproductive toxicity, including adverse effects on sperm count, morphology, motility and antioxidant enzyme activities in Wistar rats [221]. Similarly, in male Wistar rats co-administered imidacloprid (IMI), curcumin minimized reproductive toxicity parameters, oxidative stress indicators and histopathological alterations while promoting functional restoration [222].

Quercetin reversed atrazine-induced decreases in sperm count, sperm motility and daily sperm production, reduced sperm abnormalities and improved the balance between pro-oxidant and antioxidant systems in rats [223].

Naringenin improved testicular weight, epididymal sperm count and serum testosterone levels in male rats exposed to permethrin [224].

Myricetin (MYR) mitigated atrazine-mediated functional disturbances in the reproductive axis through antioxidant and anti-inflammatory mechanisms and improved epididymal sperm count and motility while reducing sperm abnormalities in atrazine-exposed rats [225].

Mice treated with allicin, a bioactive compound derived from garlic, and hesperidin (HSD), a citrus flavonoid, showed reductions in abnormal sperm morphology and DNA damage together with improvements in sperm motility, viability and membrane integrity compared with animals exposed to p-nonylphenol alone. Following combined treatment, antioxidant enzyme activities and hormone levels increased, whereas MDA concentrations decreased. Furthermore, expression of pro-apoptotic genes Bax and caspase-3 declined while anti-apoptotic Bcl-2 expression increased, indicating that allicin and HSD alleviate p-NP-induced testicular damage through enhancement of antioxidant defenses and regulation of apoptosis [226].

Flavonoid fractions containing diosmin and HSD improved sperm concentration, motility, morphology and viability, as well as testicular glycogen content and serum FSH, LH and testosterone concentrations in chlorpyrifos-exposed rats [227].

Resveratrol mitigated atrazine-induced reproductive toxicity, including reduction in serum testosterone, upregulation of caspase-3 and inducible nitric oxide synthase (iNOS} mRNA expression, destruction of seminiferous tubules, accumulation of collagen fibres within the tunica albuginea and interstitial tissue, abnormal sperm morphology, vacuolization and mitochondrial damage in germ cells [228].

Nano-quercetin effectively counteracted triazophos-induced reproductive toxicity. Triazophos reduced sperm count, viability, motility and normal morphology, decreased the number of spermatozoa with intact acrosomes and HOST-positive spermatozoa, increased reactive oxygen species generation and activated apoptotic pathways in a dose-dependent manner. These pathological changes were substantially ameliorated by nano-quercetin co-administration [229].

Lupeol, a dietary triterpene with antioxidant properties, reversed histological and biochemical alterations as well as sperm morphological defects induced by malathion, chlorpyrifos and tebuconazole exposure [230].

Thymol co-administration significantly mitigated reproductive toxicity induced by IMI, including reductions in body and reproductive organ weights, deterioration of sperm parameters, decreases in serum testosterone, histological abnormalities, apoptosis and downregulation of steroidogenic genes [231]. Similarly, thymol ameliorated reproductive dysfunction induced by hexachlorobenzene, including reductions in body and organ weights, sperm quality, hormone levels and antioxidant enzyme activities, while decreasing oxidative stress, interleukin-1β levels and caspase-3 activity [232].

Overall, polyphenols, flavonoids and related phytochemicals consistently improve sperm quality, preserve testicular histoarchitecture, restore steroidogenesis and hormonal balance, and attenuate oxidative stress, inflammation and apoptosis induced by a broad spectrum of EDs Their multitarget mode of action makes them one of the most promising groups of natural compounds for limiting endocrine disruptor-associated male reproductive toxicity.

### 6.3. Medicinal Plant Extracts and Traditional Herbal Preparations

Medicinal plant extracts and traditional herbal preparations constitute another important group of compounds investigated for their ability to alleviate endocrine disruptor-induced reproductive toxicity. Owing to their complex phytochemical composition, these preparations often exert pleiotropic effects, including antioxidant, anti-inflammatory, anti-apoptotic and pro-steroidogenic activities, leading to improvements in spermatogenesis, testicular histology and sperm quality.

At the highest dose, *Myrtus communis* essential oil, combined with BPA, improved sperm parameters, restored testicular histology, preserved membrane phospholipid composition, and normalized levels of steroid hormones including testosterone, estradiol, progesterone, and cortisol [181].

The *Cistanche tubulosa* (CT), a traditional Chinese medicine used to boost male sexual function, and echinacoside (ECH), one of its major constituents, reversed BPA-induced abnormalities in sperm characteristics and testicular structure and normalized serum testosterone levels. This was accompanied by increased expression of LDH-x as well as key steroidogenic enzymes including StAR, CYP11A1, 3β-HSD, 17β-HSD and CYP17A1, suggesting enhanced testosterone biosynthesis [233].

*Rosa roxburghii*, an ethnic medicinal herb (RRJ), significantly improved sperm quality, alleviated testicular atrophy, restored endocrine disorders, and mitigated oxidative stress, inflammation, and apoptosis in testicular tissues induced by DEHP. RRJ also reduced testicular and sperm DNA damage, as evidenced by decreased expression of γ-H2AX, a phosphorylated histone variant that serves as a marker of DNA double-strand breaks, and a lower DNA fragmentation index [234].

The maturation arrest in germ cells of guinea pigs caused by TCDD was ameliorated by *Panax ginseng* extract, and the extent of testicular recovery was dose-dependent [235].

Pine bark extract (Pycnogenol^®^, PYC, Horphag Research Ltd., Route de Belis, 40420 Le Sen, France) treatment significantly prevented α-chlorohydrin-induced germ cell toxicity, including reduced sperm motility, histopathological lesions, and apoptotic changes in the caput epididymis. Moreover, PYC exerted antioxidant effects by regulating levels of pro- and antioxidant enzymes [236].

Ethanolic extract of *Tribulus terrestris*, administered together with alpha-cypermethrin, increased testes and epididymis weight, sperm head counts, sperm motility, live sperm counts, T, FSH, LH, GSH, CAT, SOD, GST, GR, GPx and total protein content in male Wistar rats compared with reproductive toxic effects induced by alpha-cypermethrin alone [237].

Co-administration of oats (*Avena sativa* L.) oil to deltamethrin-treated mice improved total sperm density, motility and morphology and ameliorated testicular biochemical parameters as well as histological impairments in the testes [238].

Supplementation with palm oil tocotrienol-rich fraction (TRF) attenuated the detrimental effects of fenitrothion by significantly increasing sperm count, motility, viability, SOD activity and GSH level, while decreasing abnormal sperm morphology, sperm DNA damage, and MDA and PC levels in rats [239].

Ginger, which contains phenolic acids and flavonoids, preserved sperm quality, restored hormone profiles, increased steroidogenic activity and prevented oxidative injury-promoted testicular apoptosis induced by the triazole fungicide difenoconazole [240].

*Chlorella vulgaris* improved the proportions of motile and live spermatozoa, testosterone concentration, and testicular SOD and CAT levels, while reducing the proportion of abnormal spermatozoa and testicular malondialdehyde levels in rats exposed to deltamethrin [241].

*Ferulago angulata* hydroalcoholic extract (FAE) reduced adverse effects induced by chronic diazinon exposure, including decreased sperm quality, impaired sperm chromatin maturity and integrity, increased oxidative stress and enhanced lipid peroxidation [242].

Barkallah et al. [243] showed that administration of *Spirulina* extract protected mice against deleterious effects of bifenthrin in testicular tissue by preventing alterations in antioxidant enzyme activities, reducing MDA, PCO and NO concentrations, normalizing the expression of miRNAs and mRNAs involved in spermatogenesis, and restoring testosterone production together with epididymal sperm viability and motility.

*Ginkgo biloba* extract protected against deltamethrin-induced reproductive toxicity by regulating ubiquitination- and autophagy-related mechanisms, including upregulation of S-phase kinase-associated protein 2 (SKP2), an E3 ubiquitin ligase involved in protein degradation, and suppression of Beclin1, a key initiator of autophagosome formation, thereby reducing excessive autophagy and testicular damage [244].

Date palm kernel extract ameliorated fenpropathrin-induced reproductive toxicity by reducing oxidative stress, improving sperm count and motility, restoring StAR gene expression, and partially reversing the decline in serum testosterone levels [245].

Administration of *Sambucus nigra* extract mitigated fenpropathrin-induced reproductive toxicity, including decreases in sperm count and motility, reduced testosterone levels, and impaired StAR gene expression, while restoring spermatogenesis in male rats [246].

*Nigella sativa* oil (NSO) mitigated or modulated adverse effects induced by acetamiprid, including alterations in reproductive organ weights, semen quality, testosterone concentration and thiobarbituric acid-reactive substance levels [247].

Taken together, medicinal plant extracts and traditional herbal preparations consistently improve sperm quality, testicular histology, hormonal status and antioxidant defense systems in experimental models exposed to EDs. Despite differences in phytochemical composition, most of these preparations exert their protective effects through attenuation of oxidative stress, restoration of steroidogenesis, reduction of apoptosis, and preservation of spermatogenesis.

### 6.4. Novel Bioactive Compounds and Pharmacological Interventions

In addition to antioxidants and plant-derived preparations, several novel bioactive compounds and pharmacological agents have demonstrated protective effects against endocrine disruptor-induced reproductive toxicity through modulation of specific molecular pathways involved in spermatogenesis, steroidogenesis, apoptosis, and cellular homeostasis.

Cordycepin, a bioactive nucleoside derived from *Cordyceps militaris*, dose-dependently restored average testosterone levels, improved sperm density and morphology, and recovered seminiferous epithelial thickness compared to BPA treatment alone. At the molecular level, cordycepin attenuated BPA-induced suppression of PI3K, mTOR, and hypoxia-inducible factor 1-alpha (HIF-1α) expression at both mRNA and protein levels. In vitro assays confirmed that cordycepin exerted its protective effects by activating the PI3K/mTOR/HIF-1α signaling pathway [248].

The study on male mice demonstrated that chronic DEHP exposure impaired testicular development, leading to morphological abnormalities and reduced sperm quality, which were largely restored by capsaicin, the bioactive compound found in chili peppers. Capsaicin modulated the BAX/BCL2 ratio and inhibited testicular apoptosis, restored DEHP-induced suppression of testosterone biosynthesis in Leydig cells, and maintained the integrity of the blood-testis barrier in Sertoli cells [249].

Aminoguanidine (AG) treatment reversed TCDD-induced testicular damage, including oxidative stress, histopathological alterations and deterioration of sperm parameters in rats [250].

Glycine improved testicular tissue morphology and increased sperm numbers in animals affected by IMI. Furthermore, glycine regulated the expression of key enzymes involved in testosterone biosynthesis, including P450c17, P450scc, 17β-HSD, and 3β-HSD, thereby enhancing steroidogenic activity. Glycine intervention also reduced IMI-induced oxidative stress and ameliorated spermatogenic impairment [251].

Dinotefuran (DINO) exposure decreased testicular weight, sperm count, motility and viability, increased sperm aneuploidy and caspase-3 expression, and interacted with key enzymes and receptors involved in steroidogenesis and reproductive regulation. These adverse effects were ameliorated by *Rosmarinus officinalis* extract (RE) treatment in rats [252].

Salama et al. [253] demonstrated that telmisartan alleviated fipronil-induced testicular toxicity by restoring serum T, LH, and FSH levels, improving testicular and epididymal histoarchitecture, enhancing antioxidant defenses, and normalizing the expression of mitochondrial regulatory factors, including peroxisome proliferator-activated receptor gamma coactivator 1-alpha (PGC-1α), mitofusin 2 (MFN2), and mitochondrial transcription factor A (TFAM). These effects were accompanied by reduced malondialdehyde (MDA) levels and downregulation of dynamin-related protein 1 (DRP1), suggesting protection against mitochondrial dysfunction induced by fipronil exposure.

### 6.5. Microbiota-Related and Probiotic Approaches

Modulation of the microbiota has recently emerged as a promising strategy for mitigating endocrine disruptor-induced reproductive toxicity.

The study of Zhu et al. [254] reported that *Lactiplantibacillus plantarum* (RS20D) mitigated histological damage to the testes caused by MBP or DBP, restored sperm concentration, reduced morphological abnormalities, and improved the proliferation index in MBP-exposed rats. Moreover, RS20D partially reversed spermatogenic damage in DBP-exposed rats. The probiotic restored serum levels of estradiol and testosterone as well as superoxide dismutase activity in DBP-exposed rats, elevated testosterone levels in MBP-exposed animals, and normalized copper concentrations in the testes following exposure to DBP or MBP. Interestingly, RS20D also modulated gut microbial composition and partially alleviated phthalate-induced dysbiosis, suggesting that restoration of intestinal homeostasis may contribute to its protective effects on male reproductive function.

These findings are consistent with the emerging concept of the gut-testis axis, which proposes a functional link between intestinal microbiota and male reproductive health. Increasing evidence indicates that gut microbiota may play an important role in male reproductive function through the so-called gut–testis axis. Alterations in microbiota composition may contribute to endocrine disruption, oxidative stress, inflammation, impairment of blood–testis barrier integrity and dysregulation of steroidogenesis. Consequently, probiotic and microbiota-targeted interventions have recently emerged as promising strategies for mitigating endocrine disruptor-induced reproductive toxicity, although evidence remains limited and further studies are required [255,256]. Although evidence in this area remains limited, these findings suggest that probiotic-based interventions may represent a novel therapeutic avenue for reducing endocrine disruptor-induced reproductive dysfunction through modulation of redox balance, endocrine homeostasis and testicular function. Collectively, the available data suggest that microbiota-based therapies may represent a novel approach for reducing endocrine disruptor-induced reproductive dysfunction through the maintenance of redox balance, endocrine homeostasis, and testicular function.

### 6.6. Common Mechanisms of Protective Action

Although the compounds described above differ substantially in their origin, chemical structure, and molecular targets, their protective effects converge on several common biological pathways.

Taken together, the presented findings suggest that numerous compounds are capable of mitigating the adverse effects of EDs on the male reproductive system. Despite differences in their origin and molecular structure, most exert their protective effects through similar pathways involving attenuation of oxidative stress, reduction of apoptosis, improvement of antioxidant capacity, restoration of hormonal balance and steroidogenesis, and protection of spermatogenesis. As a consequence, improvements in sperm count, motility, morphology, testicular histology, and reproductive hormone levels have been observed in many experimental models.

The most consistently reported mechanism involves the attenuation of oxidative stress through the enhancement of endogenous antioxidant defense, including increased activities of SOD, CAT, GPx, GST, and glutathione-related pathways, accompanied by decreased levels of LPO products and reactive oxygen species. Many compounds also suppress apoptosis through modulation of the Bax/Bcl-2 balance, inhibition of caspase activation, and preservation of germ cell survival.

Another frequently observed effect is the restoration of steroidogenic function. Numerous protective agents normalize testosterone production by regulating the expression of steroidogenic enzymes such as StAR, CYP11A1, CYP17A1, 3β-HSD, and 17β-HSD, thereby improving endocrine homeostasis. In parallel, several interventions preserve the integrity of the blood-testis barrier, maintain normal seminiferous tubule architecture, and promote germ cell proliferation and differentiation.

In addition to these common mechanisms, some compounds exert their protective actions through more specific pathways, including modulation of PI3K/mTOR/HIF-1α signaling, regulation of Nrf2/HO-1 and SIRT1/Forkhead box protein O1 (FOXO1) pathways, preservation of mitochondrial function, inhibition of autophagy-associated damage, and regulation of inflammatory signaling cascades.

Emerging evidence also suggests that modulation of the gut microbiota may contribute to protection against endocrine disruptor-induced reproductive toxicity through the gut-testis axis, influencing endocrine function, oxidative balance, and inflammatory responses.

Nevertheless, since most studies have been performed in animals or in vitro systems, further investigations are required to determine whether these beneficial effects can be translated into clinical practice. Future studies should also evaluate the efficacy of these interventions under conditions of realistic human exposure to complex mixtures of endocrine disruptors rather than single compounds.

## 7. Conclusions

Endocrine disruptors constitute a diverse group of chemicals that differ in their sources, structures, and primary molecular targets. Nevertheless, increasing evidence indicates that many of their adverse effects on the male reproductive system are mediated through a relatively limited number of interconnected mechanisms, including oxidative stress, mitochondrial dysfunction, hormonal imbalance, and epigenetic alterations. The interaction between these processes contributes to impaired spermatogenesis, defective sperm function, and disturbances in testicular steroidogenesis.

Particular attention should be paid to the effects of EDs on the male germline. Current findings indicate that exposure to these compounds may alter DNA methylation patterns, histone modifications, and non-coding RNA profiles, suggesting that the consequences of endocrine disruptor exposure may extend beyond directly exposed individuals. Although the mechanisms underlying these effects are not yet fully understood, available data support the need for further research on potential transgenerational consequences.

The studies discussed in this review also indicate that oxidative stress represents one of the most common mechanisms involved in endocrine disruptor-induced reproductive toxicity. This may explain why various antioxidants, micronutrients, and plant-derived bioactive compounds have demonstrated protective effects in experimental models despite substantial differences in their chemical properties and molecular targets. However, most of the available evidence originates from animal and in vitro studies, and its translation into clinical practice requires further investigation.

Taken together, the current evidence highlights the complexity of endocrine disruptor action and the need for a better understanding of the interactions between oxidative stress, endocrine signaling, and epigenetic regulation. Future studies should focus on long-term and transgenerational effects, realistic exposure scenarios involving mixtures of endocrine disruptors, and the development of effective preventive and therapeutic strategies.

## Figures and Tables

**Figure 1:**
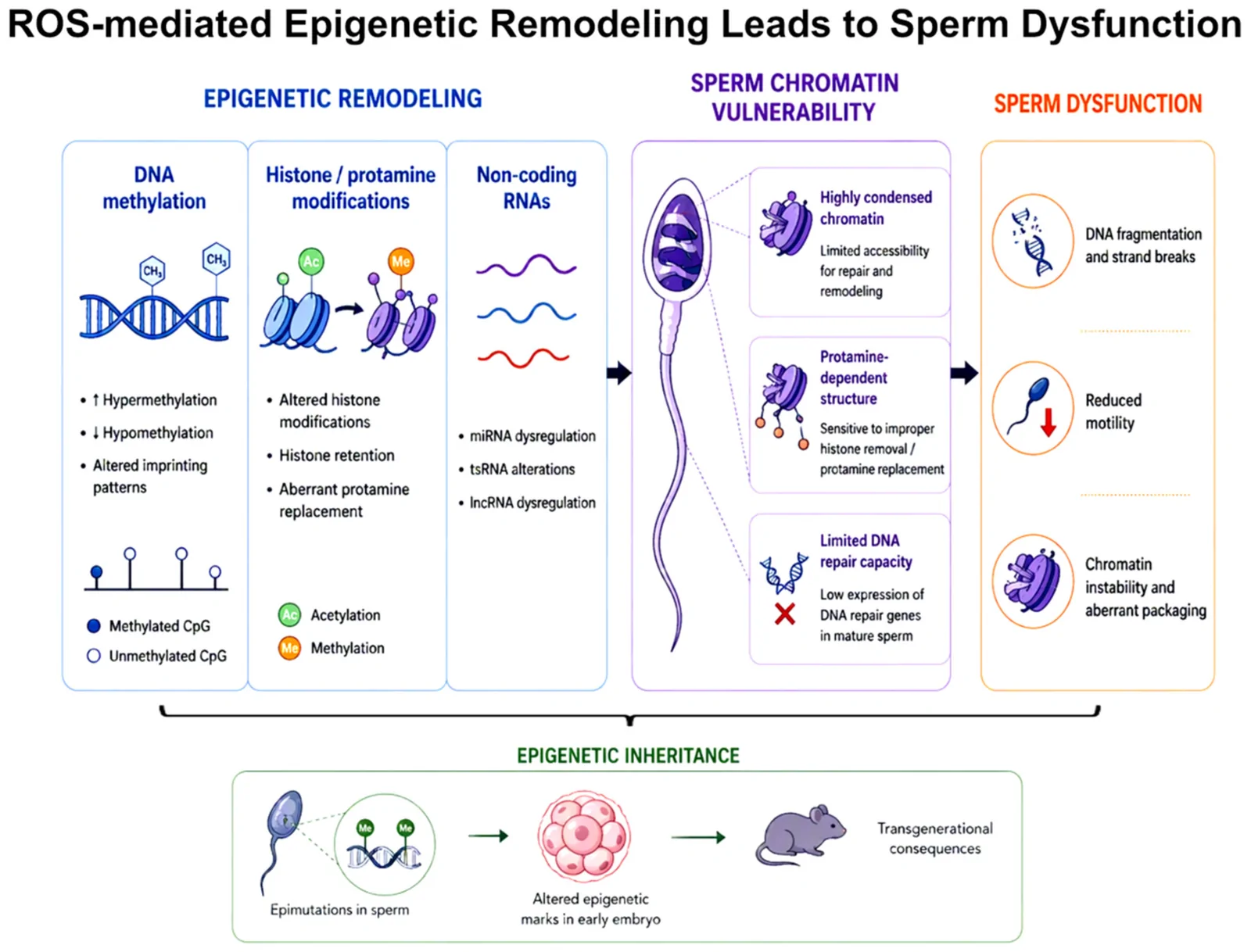
ROS-mediated epigenetic remodeling leads to sperm dysfunction and epigenetic inheritance. ROS-induced epigenetic remodeling contributes to sperm dysfunction. Oxidative stress alters DNA methylation, histone/protamine modifications, and non-coding RNA profiles, increasing sperm chromatin vulnerability characterized by high condensation and limited DNA repair capacity. These changes result in DNA fragmentation, reduced motility, and chromatin instability. Epigenetic alterations in sperm may be transmitted to the embryo, potentially leading to transgenerational effects. Abbreviations: ROS, reactive oxygen species; DNA, deoxyribonucleic acid; CpG, cytosine–phosphate–guanine dinucleotide; CH3, methyl group; Ac, acetylation; Me, methylation; miRNA, micro-RNA; tsRNA, transfer RNA-derived small RNA; lncRNA, long non-coding RNA.

**Figure 2:**
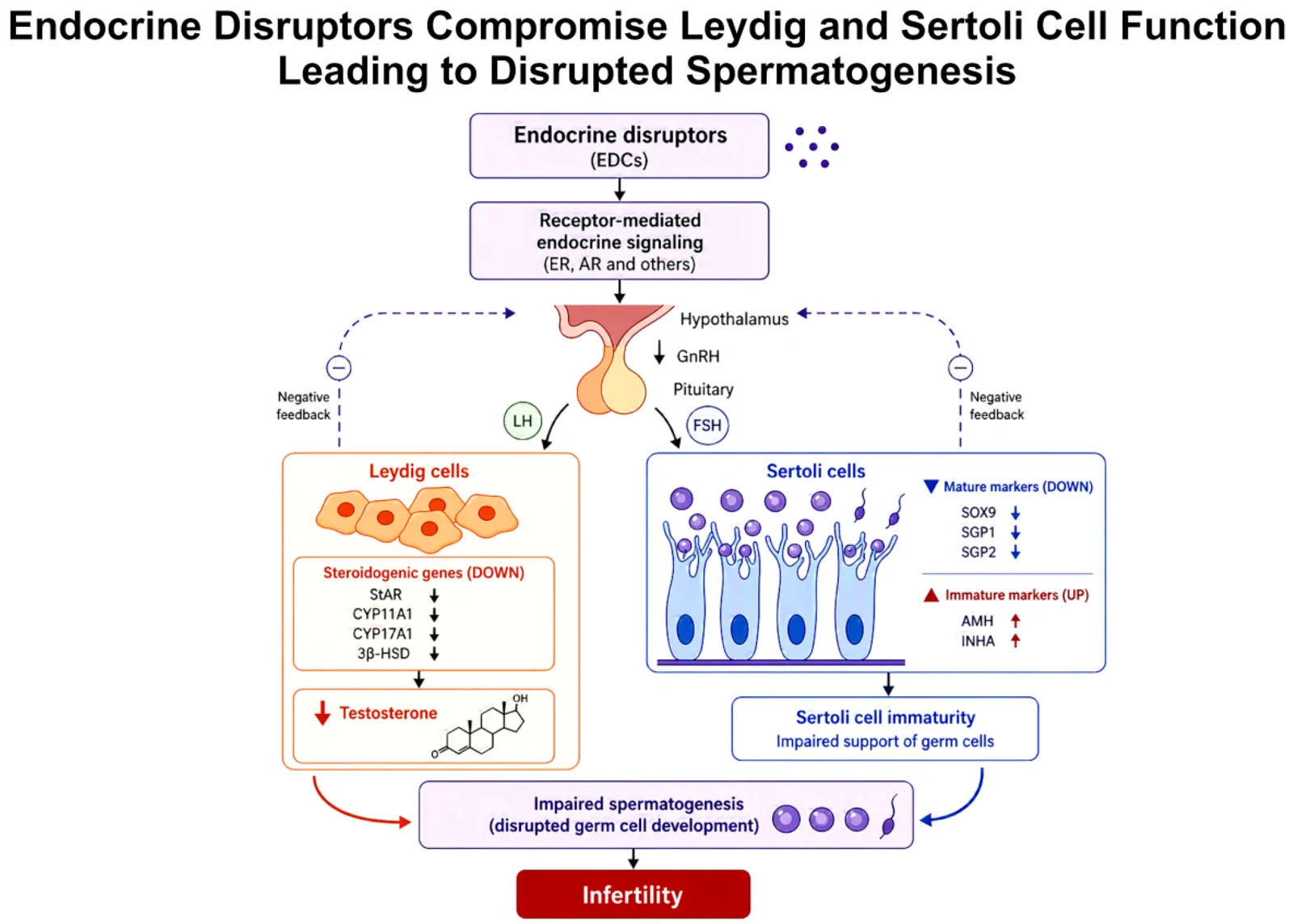
Endocrine disruptors compromise Leydig and Sertoli cell function, leading to disrupted spermatogenesis. Endocrine disruptors (EDs) impair male fertility by disrupting HPG axis signaling and testicular function. EDs alter LH and FSH signaling and directly affect testicular cells. In Leydig cells, downregulation of steroidogenic genes reduces T production. In Sertoli cells, a shift toward an immature phenotype (↓ SOX9, SGP1, SGP2; ↑ AMH, INHA) leads to impaired support of germ cells. These changes collectively result in disrupted spermatogenesis and infertility. Abbreviations: HPG, hypothalamic–pituitary–gonadal; EDs, endocrine disruptors; LH, luteinizing hormone; FSH, follicle-stimulating hormone; StAR, steroidogenic acute regulatory protein; CYP11A1, cholesterol side-chain cleavage enzyme; CYP17A1, cytochrome P450 17A1; 3β-HSD, 3β-hydroxysteroid dehydrogenase; SOX9, SRY-box transcription factor 9; SGP1, Sertoli cell glycoprotein 1; SGP2, Sertoli cell glycoprotein 2; AMH, anti-Müllerian hormone; INHA, inhibin alpha.

**Figure 3:**
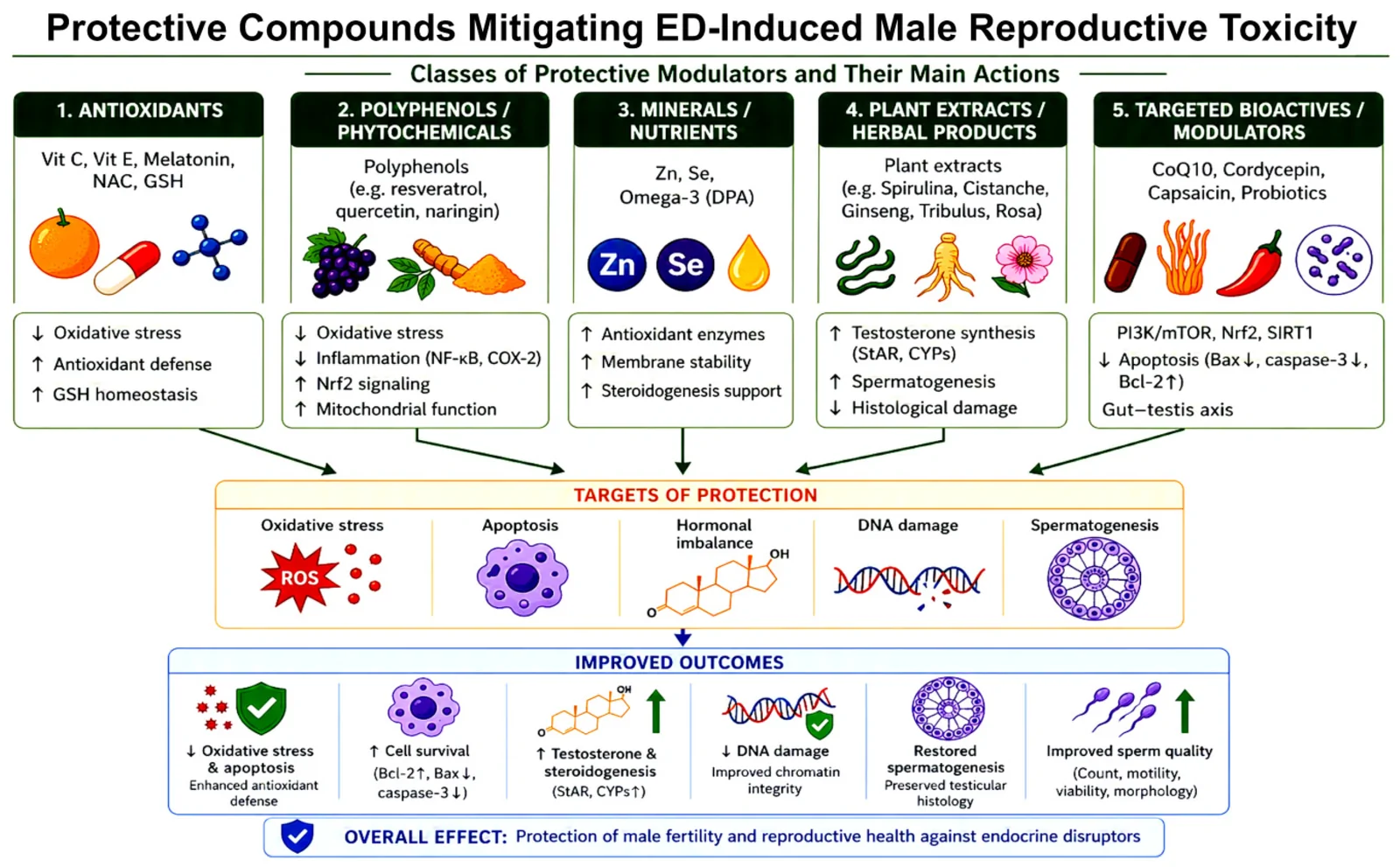
Protective compounds mitigating endocrine disruptor (ED)-induced male reproductive toxicity. Representative classes of protective modulators, including antioxidants, polyphenols/phytochemicals, minerals/nutrients, plant extracts, and targeted bioactives, attenuate ED-induced testicular toxicity. These compounds act primarily by reducing oxidative stress, inhibiting apoptosis, restoring hormonal balance, and preserving DNA integrity and spermatogenesis. Mechanistically, their effects involve enhancement of cellular antioxidant defenses, modulation of survival signaling pathways, and support of steroidogenic activity, ultimately leading to improved testicular function and sperm quality. Abbreviations: ROS, reactive oxygen species; MDA, malondialdehyde; TBARS, thiobarbituric acid reactive substances; SOD, superoxide dismutase; CAT, catalase; GPx, glutathione peroxidase; GSH, glutathione; NF-κB, nuclear factor kappa B; COX-2, cyclooxygenase-2; Nrf2, nuclear factor erythroid 2–related factor 2; SIRT1, sirtuin 1; PI3K, phosphatidylinositol 3-kinase; mTOR, mechanistic target of rapamycin; Bax, Bcl-2-associated X protein; Bcl-2, B-cell lymphoma 2 protein; Caspase-3, cysteine-aspartic protease-3; StAR, steroidogenic acute regulatory 8protein; CYPs, cytochrome P450 enzymes; DPA, docosapentaenoic acid.

## Data Availability

No new data were created in this study. Data sharing is not applicable to this article.

## Abbreviations

The following abbreviations are used in this manuscript:

8-OHdG	8-hydroxy-2′-deoxyguanosine
11β-HSD1	11β-hydroxysteroid dehydrogenase type 1
17β-HSD	17β-hydroxysteroid dehydrogenase
3β-HSD	3β-hydroxysteroid dehydrogenase
Ac	Acetylation
ACP	Acid phosphatase
AhR	Aryl hydrocarbon receptor
ALP	Alkaline phosphatase
AMH	Anti-Müllerian hormone
Bax	Bcl-2-associated X protein
Bcl-2	B-cell lymphoma 2
BHPF	Fluorene-9-bisphenol
BPs	Bisphenols
BPA	Bisphenol A
BPAF	Bisphenol AF
BPAP	Bisphenol AP
BPB	Bisphenol B
BPF	Bisphenol F
BPP	Bisphenol P
BPS	Bisphenol S
BPZ	Bisphenol Z
Caspase-3	Cysteine-aspartic protease 3
CHOP	C/EBP homologous protein
CH3	Methyl group
CHr	Chrysin
COX-2	Cyclooxygenase-2
CpG	Cytosine–phosphate–guanine dinucleotide
CYP	Cytochrome P450
CYP11A1	Cholesterol side-chain cleavage enzyme
CYP17A1	Cytochrome P450 17A1
DEP	Diethyl phthalate
DEHP	Di(2-ethylhexyl) phthalate
DETP	Diethylthiophosphate
DDE	1,1-dichloro-2,2-bis(p-chlorophenyl)ethylene
DDT	1,1,1-trichloro-2,2-bis(p-chlorophenyl)ethane
DDVP	Dichlorvos
DINP	Diisononyl phthalate
DIHP	Di-isoheptyl phthalate
DIBP	Diisobutyl phthalate
DINO	Dinotefuran
DNMTs	DNA methyltransferases
DPA	Docosapentaenoic acid
DRP1	Dynamin-related protein 1
EA	Ellagic acid
ECH	Echinacoside
ECHA	European Chemicals Agency
ED	Embryonic days
EDs	Endocrine disruptors
EFSA	European food safety authority
EOMC	*Myrtus communis* essential oil
ERα	Estrogen receptor-α
ERβ	Estrogen receptor-β
ES	Apical ectoplasmic specialization
ESAE	*Eruca sativa* aqueous extract
FAE	*Ferulago angulata* hydroalcoholic extract
FCM	Food contact materials
FOXO1	Forkhead box protein O1
FSH	Follicle-stimulating hormone
GA	Gallic acid
GMO	Genetically modified organism
GPx	Glutathione peroxidase
GR	Glutathione reductase
GRP-78	78 kDa glucose-regulated protein
GST	Glutathione S-transferase
H_2_O_2_	Hydrogen peroxide
H2AFX	H2A histone family member X
HIF-1α	Hypoxia-inducible factor 1-alpha
HO-1	Heme oxygenase-1
HOST	Hypo-osmotic swelling test
HPG	Hypothalamic–pituitary–gonadal
HSD	Hesperidin
IMI	Imidacloprid
INHA	Inhibin alpha
iNOS	Inducible nitric oxide synthase
Keap1	Kelch-like ECH-associated protein 1
LC	Lycopene
LH	Luteinizing hormone
LncRNA	Long non-coding RNA
LPO	Lipid peroxidation
MDA	Malondialdehyde
Me	Methylation
MFN2	Mitofusin 2
MRN	Flavone morin
mTOR	Mechanistic target of rapamycin
miRNA	Micro-RNA
MYR	Myricetin
NAC	N-acetyl-l-cysteine
NF-κB	Nuclear factor kappa B
NG	Naringin
NOB	Nobiletin
Nrf2	Nuclear factor erythroid 2–related factor 2
NP	Nonylphenol
NPs	Nonylphenols
PAEs	Phthalate esters
PBDEs	Polybrominated diphenyl ethers
PCBs	Polychlorinated biphenyls
PCDDs	Polychlorinated dibenzodioxins
PCDEs	Polychlorinated diphenyl ethers
PCNA	Proliferating cell nuclear antigen
PCT	Polychlorinated terphenyls
PENA	Perfluorononanoic acid
PFAS	Polyfluoroalkyl substances
PFHxS	Perfluorohexane sulfonic acid
PFOA	Perfluorooctanoic acid
PFOS	Perfluorooctane sulfonic acid
PGC-1α	Peroxisome proliferator-activated receptor gamma coactivator 1-alpha
PHB	Parahydroxybenzoic acid esters
PHBA	4-hydroxybenzoic acid
PI3K	Phosphatidylinositol 3-kinase
POPs	Persistent organic pollutants
PTMs	Post translational modifications
PVC	Polyvinyl chloride
REACH	Registration, evaluation, authorization, and restriction of chemicals
ROS	Reactive oxygen species
RRJ	*Rosa roxburghii*
RS20D	*Lactiplantibacillus plantarum*
SCCS	Scientific committee on consumer safety
SGOT	Serum glutamic-oxaloacetic transaminase
SGP1	Sertoli cell glycoprotein 1
SGP2	Sertoli cell glycoprotein 1
SGPT	Serum glutamic pyruvic transaminase
SIRT1	Sirtuin 1
SKP2	S-phase kinase-associated protein 2
SN	*Sambucus nigra*
SOD	Superoxide dismutase
SOX9	SRY-box transcription factor 9
tsRNA	Transfer RNA-derived small RNA
StAR	Steroidogenic acute regulatory protein
T	Testosterone
TBARS	Thiobarbituric acid reactive substances
TCDD	2,3,7,8-tetrachlorodibenzo-p-dioxin
TFAM	Mitochondrial Transcription Factor A
TDI	Tolerable daily intake
TDS	Testicular dysgenesis syndrome
TRF	Tocotrienol-rich fraction
TSSs	Transcription start sites
WHO	World health organization
ZGA	Zygotic genome activation
